# Biochemical properties of thyroid peroxidase (TPO) expressed in human breast and mammary-derived cell lines

**DOI:** 10.1371/journal.pone.0193624

**Published:** 2018-03-07

**Authors:** Marlena Godlewska, Wanda Krasuska, Barbara Czarnocka

**Affiliations:** Department of Biochemistry and Molecular Biology, Center of Postgraduate Medical Education, Warsaw, Poland; Duke University School of Medicine, UNITED STATES

## Abstract

Thyroid peroxidase (TPO) is an enzyme and autoantigen expressed in thyroid and breast tissues. Thyroid TPO undergoes a complex maturation process however, nothing is known about post-translational modifications of breast-expressed TPO. In this study, we have investigated the biochemical properties of TPO expressed in normal and cancerous human breast tissues, and the maturation process and antigenicity of TPO present in a panel of human breast tissue-derived cell lines. We found that the molecular weight of breast TPO was slightly lower than that of thyroid TPO due to decreased glycosylation and as suggest results of Western blot also shorter amino acid chain. Breast TPO exhibit enzymatic activity and isoelectric point comparable to that of thyroid TPO. The biochemical properties of TPO expressed in mammary cell lines and normal thyrocytes are similar regarding glycan content, molecular weight and isoelectric point. However, no peroxidase activity and dimer formation was detected in any of these cell lines since the majority of TPO protein was localized in the cytoplasmic compartment, and the TPO expression at the cell surface was too low to detect its enzymatic activity. Lactoperoxidase, a protein highly homologous to TPO expressed also in breast tissues, does not influence the obtained data. TPO expressed in the cell lines was recognized by a broad panel of TPO-specific antibodies. Although some differences in biochemical properties between thyroid and breast TPO were observed, they do not seem to be critical for the overall three-dimensional structure. This conclusion is supported by the fact that TPO expressed in breast tissues and cell lines reacts well with conformation-sensitive antibodies. Taking into account a close resemblance between both proteins, especially high antigenicity, future studies should investigate the potential immunotherapies directed against breast-expressed TPO and its specific epitopes.

## Introduction

Human thyroid peroxidase (TPO), the crucial enzyme responsible for biosynthesis of hormones by the thyroid gland, catalyzes iodination and coupling of tyrosine residues in thyroglobulin, which leads to the synthesis of triiodothyronine and thyroxine [1, 2]. TPO is also a major autoantigen in autoimmune thyroid disease (AITD). A majority of polyclonal TPO-specific antibodies (TPOAbs) present in sera of AITD patients react with epitopes located on two discontinuous, three-dimensional integrity-dependent immunodominant regions (IDR) on the surface of the TPO molecule, termed A and B (IDR-A and–B) [3–5]. These regions have been detected both in antigenic competition experiments with a panel of murine monoclonal antibodies (mAbs) [6] and using recombinant human Fab fragments [7, 8].

TPO, together with myeloperoxidase (MPO), lactoperoxidase (LPO) and eosinophil peroxidase (EPO), belongs to the family of heme-containing human peroxidases. The human *TPO* gene is located on chromosome 2 and encodes a 933-amino acid protein. The mature TPO protein has a molecular weight of approximately 100 kDa and consists of a large N-terminal extracellular ectodomain followed by short transmembrane and cytoplasmic regions. The ectodomain, exposed to the lumen of thyroid follicles, is composed of an N-terminal signal peptide, a propeptide, and the following subsequent domains: N-terminal MPO-like domain, complement control protein (CCP)-like domain, and epidermal growth factor (EGF)-like domain. During intracellular trafficking to the cell membrane, TPO undergoes several post-translational modifications, such as proteolytic trimming, glycosylation, heme fixation, and finally dimerization. Newly synthesized TPO molecules undergo core glycosylation and the heme incorporation in the membrane of the endoplasmic reticulum [9, 10], and the oligosaccharides of the TPO molecules are further modified while being transported via the secretory pathway [10]. The N-terminal propeptide is removed after exiting the Golgi apparatus complex but before the molecules reach the cell membranes [11]. The processes of TPO dimerization and the homodimer organization are rather poorly understood. However, one molecular modeling study provided structural insight to the dimerization of TPO molecules [12]. Interestingly, it suggested that only TPO dissociated into monomers is fully accessible for autoantibodies [12]. The TPO protein maturation and trafficking require the assistance of thyrocyte endoplasmic reticulum chaperones: calreticulin, calnexin and BiP [13, 14].

Several studies have reported increased levels of TPO antibodies in breast carcinoma patients [15–19]. Some authors suggested that patients with high levels of TPO-specific antibodies have a better prognosis [17, 18, 20] due to a decreased frequency of distant metastases [21]. In yet another study, a high level of TPO-directed antibodies has been shown to be associated with a lower risk of breast cancer [22]. There are also studies in which no influence of TPO antibodies on breast cancer patients’ outcome was observed [15]. Recent long-term study conducted on a largest up to date cohort of patients with moderate-to-high-risk early breast cancer did not found the TPOAbs presence as a predictive factor of the long-term recurrence and mortality [23]. The putative protective role of TPO autoantibodies in breast cancer could be explained by the fact that peritumoral and cancerous breast tissue express TPO [24, 25]. Of note, breast TPO is immunologically similar to TPO expressed in the thyroid [25]. The antigenic TPO activity depends on the proper structure of the TPO molecule. Therefore, in this study we aimed to characterize the biochemical properties of TPO expressed in human cancerous and normal mammary tissues. Additionally, we analyzed basic TPO characteristics in mammary-derived cell lines in order to determine whether these cells may be a suitable model for studies on breast TPO.

## Materials and methods

### Tissue samples

Primary breast tumor samples paired with the adjacent non-cancerous tissues were obtained from patients at different stages of breast cancer hospitalized at the Maria Skłodowska-Curie Memorial Cancer Center and Institute of Oncology (Warsaw, Poland) as described previously [25]. Archived normal human lung and Graves’ disease (G-B) thyroid tissue samples were used as controls. The study and all experimental procedures were approved by the Ethics Committee of Human Studies of the Center of Postgraduate Medical Education and at the Memorial Cancer Center and Institute of Oncology (Warsaw, Poland). Written informed consents were obtained from all patients.

### Cell lines

Chemically immortalized normal human breast epithelial cell line, 184A1, used as a non-cancerous control, and breast cancer cell lines, MCF-7 and MDA-MB-231, were obtained from American Type Culture Collection (ATCC). SV40-immortalized human thyroid epithelial line, NTHY-ori 3–1 (NTHY), used as a positive control, was purchased from European Collection of Cell Cultures. Estrogen receptor (ER)-positive MCF-7 cells were maintained in Eagle’s Minimum Essential Medium (EMEM; Sigma-Aldrich, Steinheim, Germany) supplemented with 0.01 mg/ml bovine insulin (Sigma-Aldrich) and 10% fetal bovine serum (v/v) (FBS; Roche, Mannheim, Germany). ER-negative MDA-MB-231 cells were cultured in Dulbecco’s Modified Eagle Medium (DMEM; Life Technologies, Gibco, NY, USA) supplemented with 10% FBS (Roche, Mannheim, Germany). 184A1 cells were maintained in Mammary Epithelial Cell Growth Medium (MEGM; Lonza, Walkersville, USA) supplemented with 0.005 mg/ml transferrin (Sigma-Aldrich) and 1 ng/ml of cholera toxin (Sigma-Aldrich), while NTHY cells were grown in RPMI-1640 medium (Life Technologies) supplemented with 10% FBS (Roche, Mannheim, Germany). All cell lines were cultivated at 37°C in humidified atmosphere with 5% CO_2_.

### Antibodies

A panel of four mouse anti-TPO monoclonal antibodies, i.e. mAb 47, mAb 15, mAb 18, mAb 64, were generously provided by Prof. J. Ruf from UMR-MD2, Aix-Marseille University, Marseille Medical School, Marseille, France and had been previously characterized [6, 26]. TPO-specific mAb A4 was kindly provided by Prof. J. P. Banga [27]. Rabbit antibodies to synthetic P14 peptide of TPO (aa 599–617) has been generated as already described [28–30]. TPO-specific mAb ab76935 and β-actin-specific mAb (clone AC-15) were purchased from Abcam. Archival sera were used to prepare a pool of sera with high titers of TPO-specific antibodies (n = 5) and a pool of sera free of TPOAbs (n = 5). Rabbit polyclonal antibodies 10376-1-AP specific to human lactoperoxidase (LPO) were obtained from ProteinTech (Chicago, USA) and the murine isotype IgG1 control was purchased from Dako (Glostrup, Denmark).

### Total protein isolation, thyroid peroxidase immunoprecipitation and Western blotting

To extract total proteins, the cells were washed with chilled PBS (pH 7.3) twice, then scraped into PBS and centrifuged (500 x g for 5 min at 4°C). Next, the pelleted cells were resuspended in RIPA buffer (ThermoScientific, Rochester, USA) containing cOmplete Protease Inhibitor Cocktail (Roche). After incubation on ice for 30 minutes, the samples were centrifuged at 16000 x g for 30 minutes at 4°C and the supernatants were collected. Protein extracts from human tissues were obtained as previously described [25]. Protein concentration was determined by the BCA Protein Assay Kit (ThermoScientific). Then, the protein samples were aliquoted and stored at -70°C. TPO was immunoprecipitated with rabbit antipeptide P14 serum from breast tissue lysates as previously described [25].

Protein samples were resolved on an 8% SDS-PAGE gel under reducing conditions and electrotransferred to nitrocellulose membranes (Bio-Rad, Richmond, USA). After one-hour blocking in 5% non-fat milk in TBS with 0.1% Tween 20 (TBS-Tween 0.1%), the membranes were probed with the primary antibody at 1 μg/ml (ab76935 and anti-β-actin monoclonal antibodies) or 33 ng/ml (10376-1-AP antibody) in blocking buffer overnight at 4°C. This was followed by incubation with a HRP-conjugated rabbit anti-mouse IgG (JacksonImmuno Research, West Grove, USA) diluted 1:20000–1:50000 in TBS-Tween 0.1% or with a HRP-conjugated goat anti-rabbit IgG (Dako) diluted 1:20000–1:100000 in the blocking buffer. The signals were then developed using SuperSignal West Pico Chemiluminescent Substrate and SuperSignal West Dura Extended Duration Substrate (ThermoScientific). Protein bands were analyzed using the GelAnalyser 2010a software (http://www.gelanalyzer.com). The anti-TPO ab76935 antibody was preabsorbed as described elsewhere [25].

### Deglycosylation with peptide-N-glycosidase F (PNGase F)

To analyze the asparagine-linked glycan contents in TPO, deglycosylation with PNGase F was performed. To this end, protein samples were denatured for 10 minutes in 0.5% SDS (w/v) and 40 mM dithiothreitol (DTT) at 95°C. Afterwards, sodium phosphate (pH 7.5) and Nonidet P-40 were added to the final concentrations of 50 mM and 1% (v/v), respectively. Finally, the reaction mixture was supplemented with PNGase F (New England Biolabs, Hitchin, UK) and incubated at 37°C for 16 hours. Control reactions were performed without PNGase F using the same assay conditions. Finally, the samples were analyzed by Western blotting.

### Two-dimensional electrophoresis (2-DE)

Total protein extracts (100 μg) from human tissues or cultured cells in 2-DE rehydration buffer (7 M urea, 2 M thiourea, 4% CHAPS (w/v), 2 mM tributylphosphine (TBP), 0.4% BioLyte 3–10 (v/v), cOmplete Protease Inhibitor Cocktail (Roche)) were isoelectrofocused on 7 cm pH 3–10 IPG strips using PROTEAN IEF Cell (Bio-Rad), according to the manufacturer’s instructions. Afterwards, IPG strips were incubated in the equilibration buffer (20% glycerol (v/v), 2% SDS, 6 M urea, and 50 mM Tris; pH 8.8) supplemented with 2% DTT (w/v) for 15 minutes, followed by the incubation with the fresh equilibration buffer supplemented with 2.5% iodoacetamide (w/v) for another 15 minutes. Next, the strips were washed in 25 mM Tris, 1.92 M glycine, 0.1% SDS (pH 8.3), and a standard Western blot analysis was performed. If not indicated otherwise, all equipment and reagents used were purchased from Bio-Rad.

### Detecting TPO enzymatic activity

The enzymatic activity of cell surface-expressed TPO in the studied cell lines was analyzed as we had previously described [31]. Briefly, cells were grown 48 h in a medium supplemented with 20 mM hemin (Sigma-Aldrich). After washing with PBS, cells were incubated with reaction mixture containing Amplex Ultra Red (Life Technologies) in the presence of superoxide dismutase (SOD), KI, and H_2_O_2_ (Sigma-Aldrich). Reaction was stopped at 1-minute intervals by addition of catalase and SOD. The fluorescence was measured in the Synergy H4 hybrid multi-mode microplate reader (BioTek, USA) [31]. The enzymatic activity of TPO expressed in tissues was determined using a luminol-based assay described by Jomaa and collaborators [25, 32], with some modifications. Briefly, breast tissue lysates (200 μg) were immunoprecipitated with mAb A4 (6 μg) and protein A agarose (Merck Millipore, Darmstadt, Germany). 50 μl of resin-bound TPO in 0.1 M Tris-Cl (pH 8.6) was incubated with 150 μl of 1.3 M glycine-NaOH (pH 9.0), 1.3 mM EDTA in a 96-well plate for 5 minutes. The reaction was initiated by adding 20 μl of 400 μM luminol (Sigma-Aldrich) in 1 M glycine-NaOH (pH 9.0), 1 mM EDTA, followed by the supplementation with 5 μl of 80 mM H_2_O_2_. Luminescence intensities were measured in the Synergy 2 instrument (BioTek).

### Immunocytochemistry

Cells grown on uncoated glass coverslips for two days were washed with cold PBS (pH 7.3), fixed and permeabilized with methanol, then washed again and blocked in 2% goat serum (v/v) and 2% BSA (w/v) in PBS-0.1% Tween 20 (v/v) for one hour. This was followed an overnight incubation with the primary antibody at 4°C. Monoclonal antibodies were used at the concentration of 14 μg/ml, while the pooled human sera were diluted at 1:10, and the concentration of 10376-1-AP Ab was 0.4 μg/ml. Following the washing, coverslips were incubated with the goat anti-mouse IgG DyLight 549-conjugate (JacksonImmuno Research), goat anti-human IgG fluorescein isothiocyanate-conjugate (Sigma-Aldrich) or goat anti-rabbit IgG Alexa 488-conjugate (Life Technologies). Then, the cells were washed, stained with 4′,6-diamino-2-phenylindole (DAPI), mounted in Fluorescence Mounting Medium (Dako), and finally visualized using the LSM800 confocal microscope with the ZEN 2.1 software (Zeiss, Germany). The reactivity without primary antibody was determined as a negative control. To reduce non-specific binding, the concentration of Tween 20 was increased to 1% (v/v) in all solutions in the experiments with the human sera. If not indicated otherwise, the experimental steps were performed at room temperature.

### Immunohistochemistry

Immunohistochemical staining analysis was performed on 4-μm thick sections of formalin-fixed, paraffin-embedded tissues. Dewaxed, deparaffinized and rehydrated sections were incubated with target retrieval solution (pH 9.0) (Dako) at 98°C for 20 minutes. Following a 15-minute incubation with 0.3% hydrogen peroxide, tissue sections were then incubated with polyclonal anti-LPO antibody (10376-1-AP, 0.3 μg/ml) in the antibody diluent (Dako) for one hour in a humid chamber. This was followed by the incubation with REAL EnVision Detection System (Dako) for 30 minutes. Between all these steps, the sections were intensively washed in TBS-1% Tween 20. The reaction product was developed with 3,3’-diamino-benzidine (DAB; Dako) for 15 minutes. After counterstaining with hematoxylin, the sections were mounted on glass slides and examined under a light microscope (Olympus BX41, Japan). Two independent observers evaluated the immunohistochemical staining and scored it as negative or positive. All steps were performed at room temperature unless stated otherwise.

## Results

### TPO protein maturation in human breast tissue samples

**Fig 1:**
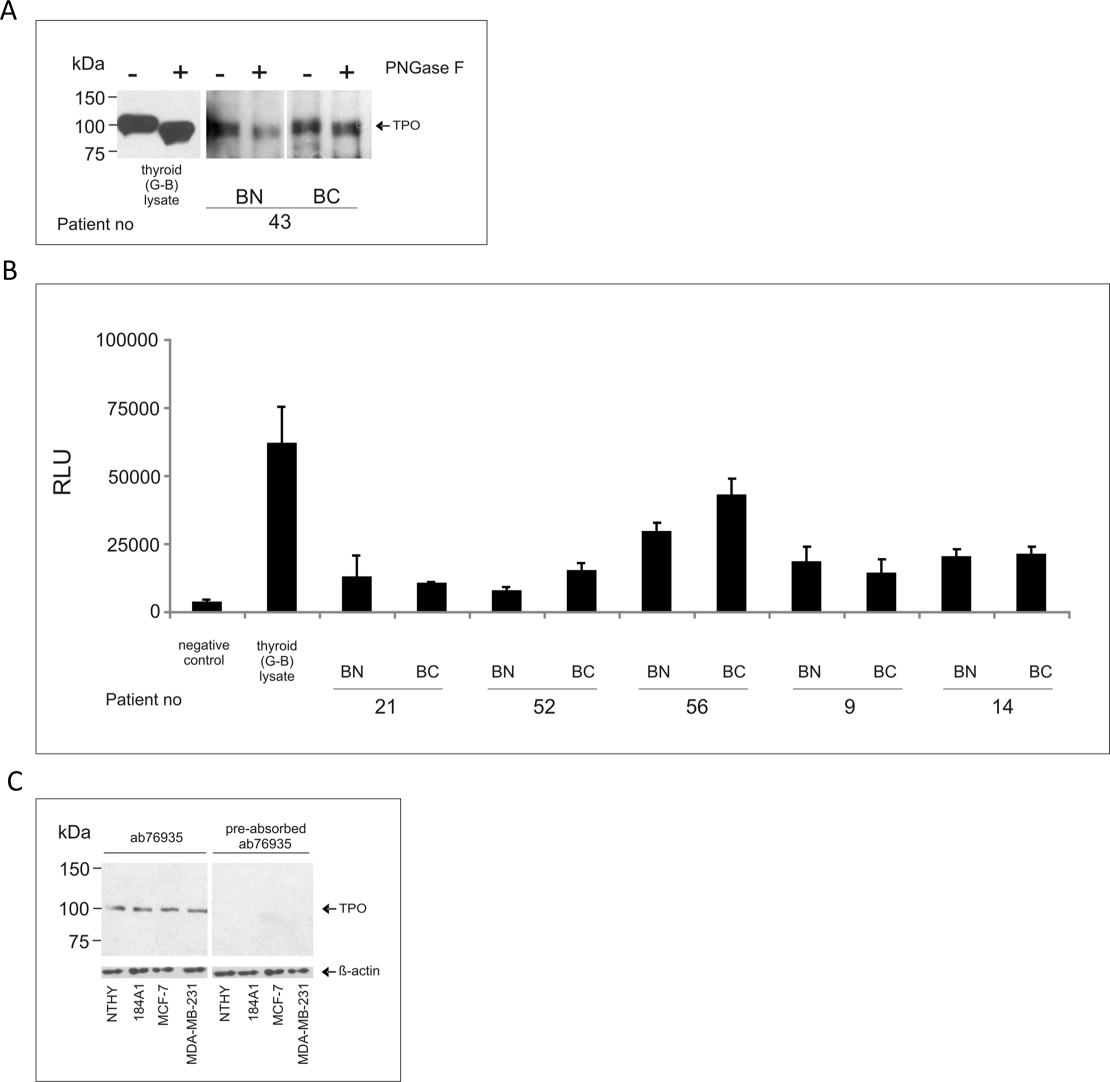
**The biochemical properties of TPO protein expressed in breast tissues (A and B) and breast-derived cell lines (C).** (A) N-linked glycan content in TPO expressed in breast tissues. Total cell extract was digested with PNGase F, then subjected to 8% SDS-PAGE, followed by Western blotting, and probing with TPO-specific mAb 47 monoclonal antibody. Controls were processed under the same conditions as the samples except that no enzyme was added. One representative immunoblot out of at least three independent experiments is shown. (B) Enzymatic activity of TPO expressed in breast tissues. Tissue lysate was incubated with TPO-specific mAb A4, then protein A agarose was added to precipitate immune complexes. TPO-antibody complexes bound to agarose were incubated with luminol in the presence of hydrogen peroxide. The intensity of luminescencent signal was measured and results were expressed as relative light units (RLU). As positive control, TPO immunoprecipitated from human thyroid tissue lysate (Graves’ disease case) was used to measure luminol oxidation. Agarose A incubated with mAb A4 alone (lysate omitted) was used as negative control. One representative of three independent experiments is shown. (C) TPO protein expression in breast epithelial normal (184A1) and cancer cell lines (MCF-7 and MDA-MB-231). Western blotting was used to detect TPO protein presence. The specificity of the reaction was verified by preabsorption of ab76935 antibody with the excess of highly purified human TPO. NTHY was used as a positive control. β-actin-specific Ab was used as a loading control. BN: peri-tumoral breast tissue; BC: breast cancer tissue; G-B: Graves’ disease thyroid tissue; NTHY: NTHY-ori 3–1 cell line; PNGase F: Peptide-N-Glycosidase F; RLU: relative light units.

We analyzed whether breast TPO undergoes similar post-translational modifications as TPO expressed in the thyroid gland. We found that the molecular weight of detected breast tissue-expressed TPO was about 94 kDa. This is consistent with our previous findings [25] and it is lower than the molecular weight of TPO in the thyroid gland (about 100 kDa). In order to further elucidate the reason for this difference, we removed all N-linked glycans and compared the shift in the migration of digested and non-digested TPO bands (Fig 1A). This revealed that the content of asparagine-linked carbohydrates in breast tissue TPO was lower (3 kDa) than in thyroid TPO (at least 10 kDa). Two-dimensional electrophoresis showed that the band containing breast tissue TPO consisted of two proteins with different isoelectric points (pIs; 6.4 ± 0.2 and 7.1 ± 0.1) which correspond to those previously described for the thyroid TPO [33]. Moreover, we observed the peroxidase activity in crude lysates of breast tissues using the guaiacol oxidation-based method. To determine whether the detected enzymatic activity is specific for TPO, we performed the peroxidase activity measurement using immunoprecipitated TPO, luminol, and H_2_O_2_. Resins incubated with the A4 monoclonal antibody and protein lysate of human thyroid tissue from a Graves’ disease case or resins incubated with the A4 monoclonal antibody alone were used as positive and negative controls, respectively. TPO immunoprecipitated from breast tissue displayed different levels of peroxidase activity (Fig 1B). Interestingly, no dimeric form of breast TPO was observed using the non-reducing SDS-PAGE method.

### TPO protein maturation and immunological activity in breast-derived cell lines

Then, we analyzed the expression levels and the biochemical properties of TPO expressed in 184A1, MCF-7, and MDA-MB-231 cell lines using described immunological and biochemical methods [25, 31]. TPO expression was confirmed in all cell lines (Fig 1C, left panel, and Figure A in S1 Fig). The levels of the TPO protein present in the analyzed cell lines were determined semi-quantitatively with a standard curve obtained using highly purified hTPO. The average amount of TPO protein expressed in breast-derived cell lines was 6.0 ± 1.7 μg of TPO per 1 mg of crude lysate. The level of the TPO protein expression was stable over time (Figure A in S1 Fig). We found that the TPO molecular weight was similar between the mammary-derived cell lines and the control NTHY cell line (98.0 ± 0.9 kDa). To confirm the specificity of the observed bands, anti-TPO antibody ab76935 was preabsorbed by highly purified hTPO. As shown in Fig 1C (right panel), preabsorption completely inhibited the signal. We have also analyzed the isoelectric point of the TPO protein using two-dimensional electrophoresis. We found that the TPO band in the extracts obtained from mammary and thyroid gland-derived cells consisted of two proteins with different pIs (Figure B in S1 Fig). The pI values were as follows: 4.9 ± 0.1 and 7.2 ± 0.1 for 184A1 cells, 4.5 ± 0.6 and 7.0 ± 0.1 for MCF-7, and 5.6 ± 0.1 and 7.5 ± 0.1 for MDA-MB-231. Similar results were obtained for the NTHY samples (pI 4.7 ± 0.1 and 7.3 ± 0.3). The N-glycosylation level of TPO expressed in mammary-originated cell lines was comparable to that of TPO in NTHY. To find out whether TPO expressed in breast is enzymatically active, we incubated the cells with a fluorogenic substrate in the presence of hydrogen peroxide and the accumulated fluorescent reaction product was measured in real time. No reactivity was detected in any of the analyzed cell lines, including NTHY. We did not observe dimeric forms of breast TPO in SDS-PAGE under non-reducing conditions, either.

**Fig 2:**
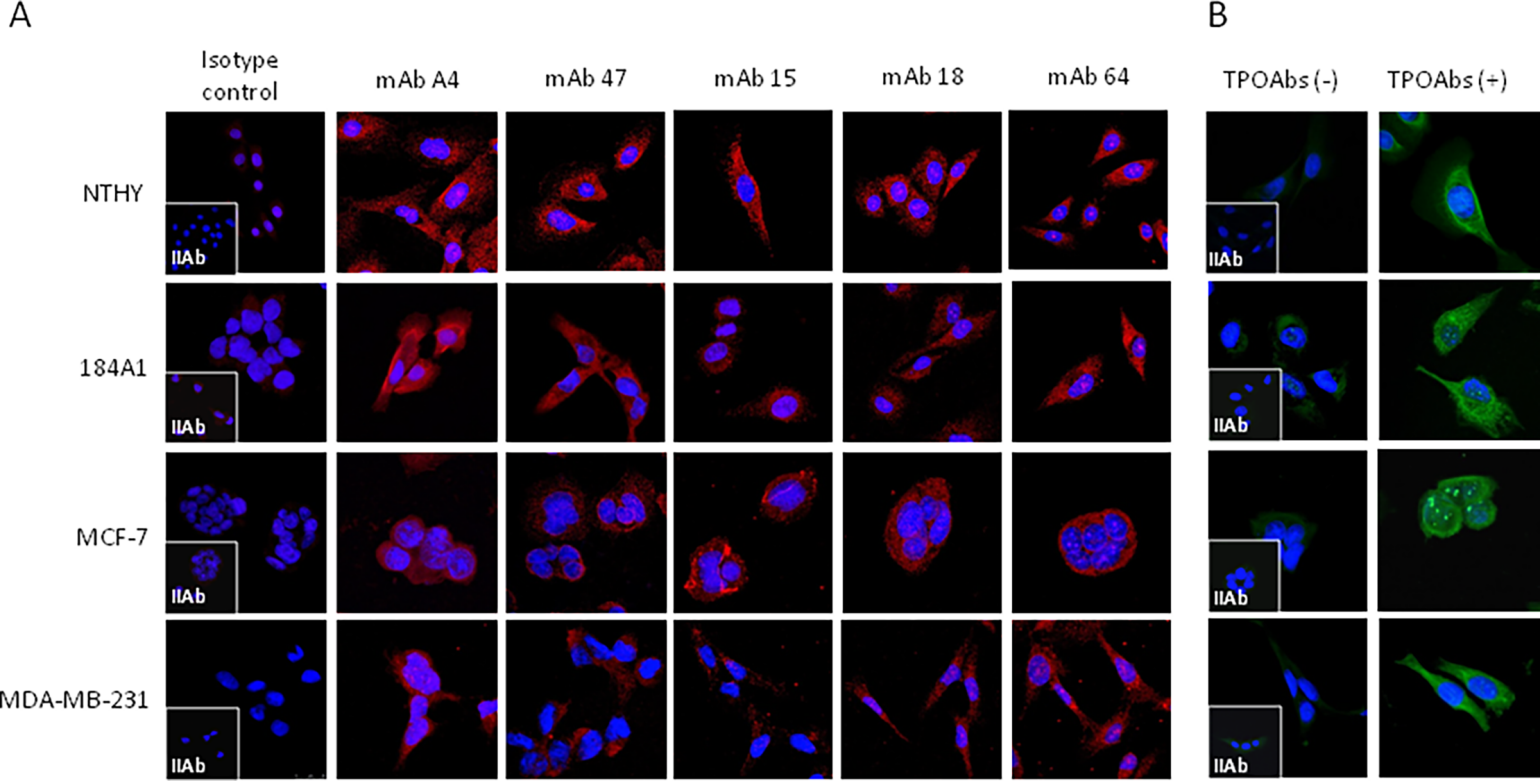
**Representative TPO immunostaining results obtained with a panel of monoclonal antibodies (mAbs) against human thyroid peroxidase (TPO) (A) and human serum pools (B) in breast epithelial normal (184A1) and cancer cell lines (MCF-7 and MDA-MB-231).** A normal human thyroid cell line, NTHY, was used as a positive control. (A) Positive signal (red) was detected with all mAbs except the isotype control (negative control). In the cells incubated with TPOAbs obtained from autoimmune thyroid disease (AITD) patients (TPOAbs(+)), a positive signal (green) was observed but this staining was not observed when TPOAb-free serum (TPOAbs(-)) was used (negative control). Nuclei (blue) were counterstained with DAPI. Magnification: 630×. IIAb: secondary antibody; DAPI: 4′,6-diamino-2-phenylindole; NTHY: NTHY-ori 3–1 cell line.

Subsequently, we evaluated the immunoreactivity of TPO expressed in the breast tissue-derived cell lines using a panel of monoclonal mouse antibodies recognizing N- (mAb A4) and C-terminal (mAb 47, 15, 18, 64) domains of human thyroid peroxidase. The specificity of mAb and human TPOAb binding was verified using a murine isotype control and a pooled human TPOAb-free serum, respectively. The labeling of TPO present in the breast cancer-derived cells, normal breast tissue-derived cells, and normal thyroid follicular cells is shown in Fig 2. No differences in TPO immunoreactivity were observed between the investigated cells in reaction with monoclonal antibodies (Fig 2A). Similar TPO staining intensity was observed between different cell lines when pooled human TPOAb-positive serum was used (Fig 2B).

### LPO protein expression in breast-originated cells and human breast tissue samples

**Fig 3:**
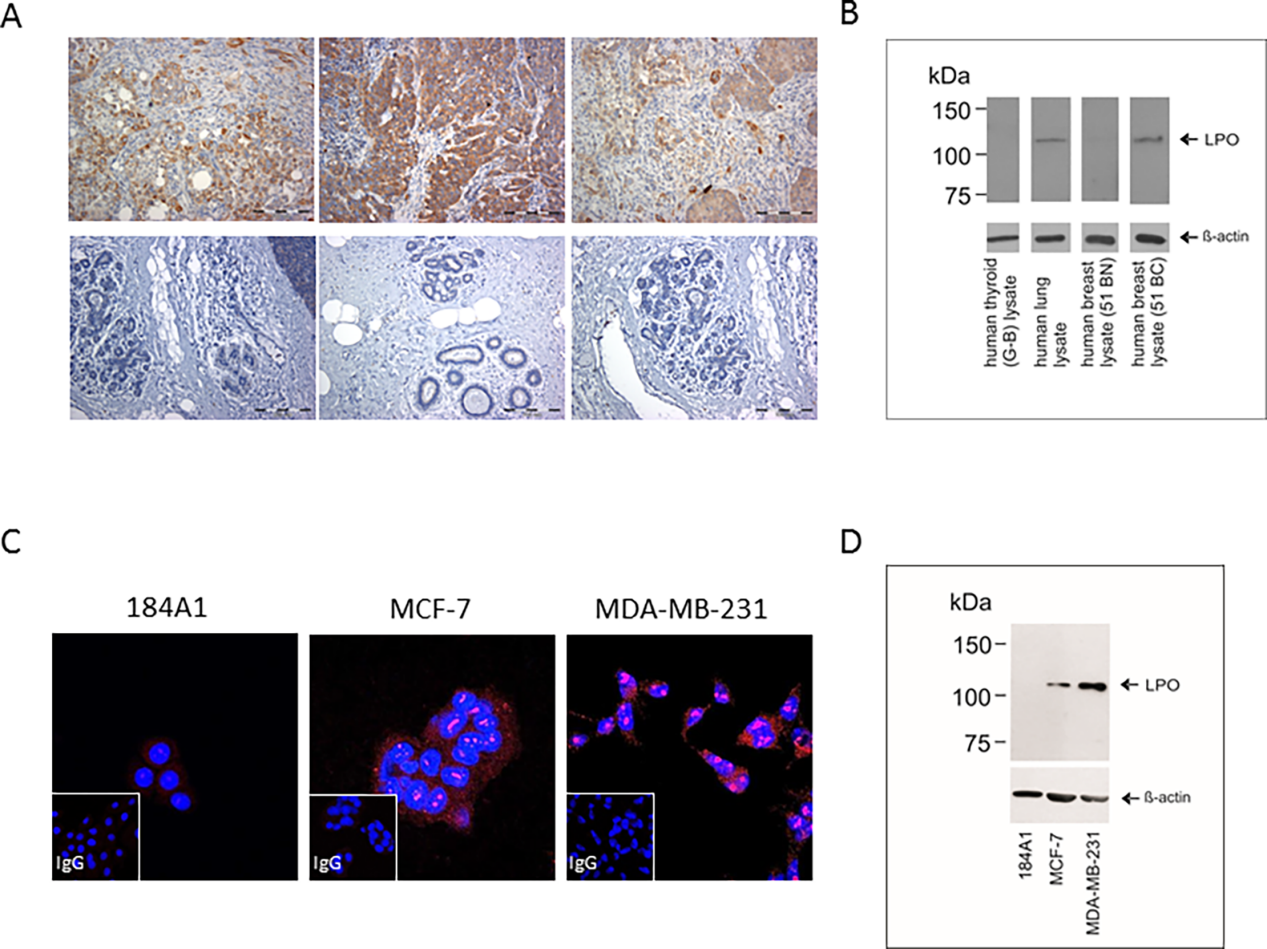
**The expression of lactoperoxidase (LPO) in breast tissues (A and B) and cell lines (C and D) derived from normal (184A1) and cancerous mammary tissues (MCF-7 and MDA-MB-231).** (A) Representative immunohistochemical staining of human LPO in breast cancer (upper panel) and peritumoral tissues (lower panel) (magnification: 100×). (B) Human LPO expression in breast cancer and peritumoral tissues detected by immunoblotting with the 10376-1-AP antibody. Human lung and thyroid tissue (Graves’ disease case) lysates were used as a negative and positive control, respectively. For each lane, 50 μg of crude protein extract were loaded. A β-actin-specific antibody was used as a loading control. (C) LPO expression in cell lines as shown by immunofluorescent staining. Positive immunofluorescent signal (red) was detected in MDA-MB-231 and, to a lesser extent, in MCF-7 cells. No staining was detected when pre-immune rabbit IgG was used (insets). Nuclei were counterstained with DAPI (blue). Magnification: 630×. (D) LPO protein expression in cell lines analyzed by Western blot. LPO was detected in breast cancer cells, while no band was observed in normal 184A1 cells. For each lane, 10 μg of crude protein extract were loaded. A β-actin-specific antibody was used as a loading control.

Since lactoperoxidase (LPO), another peroxidase from the TPO family, is also expressed in breast tissue, we performed immunochemistry analyses using cancer and normal samples and anti-LPO antibody (10376-1-AP) in order to verify whether co-expression of LPO and TPO did not influence the results obtained for TPO. We found that LPO was expressed in all the tested breast cancer samples (Fig 3A, upper panel). However, the intensity of the staining differed, depending on the tissue. We did not detect any staining in peritumoral (normal) breast tissue (Fig 3A, lower panel). This is consistent with the Western blot results which showed that LPO was expressed in breast cancer tissue and in the human lung tissue used as a positive control (Fig 3B). LPO expression was not observed in peritumoral breast tissue and thyroid tissue from the Graves’ disease patients (Fig 3B). The molecular weight of the LPO band (111 kDa ± 2 kDa) was higher than that of hTPO (about 100 kDa).

We have also performed the immunofluorescent detection of LPO in breast-derived cell lines using the 10376-1-AP antibody (Fig 3C). In both breast cancer cell lines, MCF-7 and MDA-MB-231, the LPO protein was found to be localized in the cytoplasm and in the nucleolus. However, no signals were detected in normal breast cells (184A1). No labeling was observed with rabbit IgG which was used as a negative control. Western blot results revealed bands corresponding to the LPO protein in MCF-7 and MDA-MB-231 cells, while no bands were observed for the 184A1 cells (Fig 3D).

## Discussion

We have previously demonstrated that thyroid peroxidase (TPO) expressed in human mammary tissue is immunologically active [25]. In this study, we aimed to further determine the biochemical characteristics of breast TPO. We have shown that mammary TPO, like thyroid TPO, is N-glycosylated. However, the amount of carbohydrate residues attached to the breast TPO protein core is lower than in the thyroid TPO molecule. It had been already demonstrated that N-linked oligosaccharides are important in the trafficking of TPO molecule to the cell surface and in its enzymatic activity. The immunoreactivity with TPO-specific monoclonal and polyclonal antibodies, however, was not markedly affected by a removal of asparagine-linked residues from the enzyme, indicating that carbohydrate residues seem to be rather a maturation signal for TPO as a representative of transmembrane glycoprotein [10, 34, 35]. To check whether an altered protein glycosylation pattern affects mammary TPO functionality, we conducted peroxidase activity assays. The results suggest that at least a part of breast TPO molecules are enzymatically active. Therefore, it could be hypothesized that breast TPO may participate in the regulation of oxidative stress in breast tissue. It has already been shown that LPO, another enzyme from the peroxidase family which is expressed in breast tissue can oxidize natural and synthetic estrogen to catechol estrogens which react with DNA. The mutagenic adducts that are formed during this process can initiate and promote breast cancer development [36–38]. Furthermore, it has been reported that supplementation with iodine and iodide mixture inhibits the generation of DNA adducts and exerts apoptotic effect on cancerous cells in the rat dimethylbenz[a]anthracene-induced tumor model [39]. These effects were attributed to LPO. However, given the results of our current study, the enzymatically active breast TPO might also be considered as a player involved in these processes. The molecular mass of the glycan-deprived breast TPO band is slightly lower than that found for the thyroid TPO. Since in normal and diseased thyroid glands as well as in normal and cancerous breast tissue shorter forms of TPO are expressed, the difference between molecular weights of thyroid- and breast-expressed TPO molecule might be, at least partly, explained by a lack of one exon or several exons [24, 40–42]. Nevertheless, splicing out some of the exons does not always lead to significant changes in the thyroid peroxidase structure and function. For example, an isoform abundantly expressed in normal thyroid tissue, TPO4, lacking exon 14, is not only able to acquire a proper three-dimensional structure and enzymatic activity, but also reaches the cellular surface [40]. Therefore, it seems that even if some exon(s) are missing in the breast TPO, its functionality does not seem to be significantly different from that of the full-length thyroid TPO.

Since cell lines originating from human tissues are a good tool for studying and characterizing proteins, we examined whether the breast-derived cell lines can be used as a model to study breast TPO. Our results showed that in normal and breast cancer-derived cell lines (including both the non-metastatic MCF-7 cell line that retains some of differentiated epithelial features and the aggressive triple negative MDA-MB-231 cell line) the TPO protein levels were similar to those detected in the normal immortalized thyrocyte culture. Moreover, the TPO protein expression levels were stable in time. There were no significant differences in the molecular weight, the isoelectric point (pI) value and the N-glycan content between the TPO isolated from the cell lines derived from cancerous and non-cancerous breast tissue and the TPO isolated from normal thyrocytes, either. Altogether, our results suggest that TPO molecules expressed in the thyroid and in the breast have similar biochemical properties. It is also worth noting that all antibodies used in the analysis of antigenic TPO activity have bound the TPO protein in all the tested cell lines with a similar intensity and the obtained immunocytochemical staining was shown to predominantly localize to the cytoplasmic compartment. The fact that antibodies well known to specifically recognize thyroid-expressed TPO bind to the breast TPO surface indicates that the two forms of TPO have a similar conformation. This suggests that they should also have similar functions as the TPO enzymatic activity depends on the integrity of its three-dimensional structure. However, although the conformational structure of the breast TPO seems to be very similar to that of the thyroid TPO, we did not detect any enzymatic activity of the protein in the intact cells. This may be due to the fact that the fluorogenic substrate incubated with the cells was membrane-impermeable and as a consequence only the enzymatic activity of TPO localized and exposed on the cell surface could be detected. The lack of signal for the TPO enzymatic activity is probably due to the fact that the amounts of TPO bound to cell membranes were below the detection threshold of the method. Indeed, using the membrane proteins biotinylation method (non-membrane permeable sulfo-NHS-SS-biotin) which, as we had previously described, significantly enriches this fraction [25], we detected a faint TPO band with anti-TPO antibodies in all the tested cell lines, which indicates the occurrence of low levels of cell membrane-located peroxidase. Similar observations were reported in a study conducted on the CHO cells stably transfected with human TPO cDNA, which showed that this enzyme was predominantly detected intracellularly and only a small part reached the cell surface [10]. Furthermore, supplementing the culture medium with the heme precursor (20 mM hemin) which had been shown to increase the enzymatic activity of cell membrane-bound TPO [9], did not allow to detect higher levels of the protein.

Thyroid peroxidase has a high sequence homology with human LPO (hLPO), an enzyme expressed in lactating breast tissue as well as in salivary, lacrimal, tracheal and bronchial glands [43, 44]. Therefore, we considered the potential influence of LPO detection on the obtained results. We have already shown that all TPO-specific antibodies used in the present study do not cross-react with LPO [25]. Here we show the LPO protein expression in breast cancer cell lines and breast tissue samples, while the peritumoral sections and the normal breast cell line were not positive for LPO. Low or undetectable levels of LPO in the normal mammary gland and increased expression levels in breast cancer tissue has been already reported by others [39, 45, 46]. Furthermore, we found that the molecular weight of human LPO was higher than that observed for breast TPO (approximately 111 kDa versus 100 kDa). The reported hLPO molecular weight measured in human milk is about 80 kDa for the completely proteolytically processed protein, while 90 kDa band corresponds to partly processed molecules [47]. Therefore, the hLPO band that we detected in crude protein lysates isolated from breast tissues and breast-derived cell lines may represent the beginning of the post-translational maturation of hLPO.

In conclusion, we found that the biochemical properties of the TPO protein expressed in cancer and normal breast tissue are similar to those observed for the thyroid TPO. Nevertheless, there are some differences in comparison with the thyroid-tissue expressed protein, such as a lower N-glycan content, a slightly smaller polypeptide length, a decreased enzymatic activity, and undetectable dimer formation. We also show that biochemical characteristics of the TPO protein expressed in normal (184A1) and cancerous cell lines (MCF-7 and MDA-MB-231), independently of the cancer differentiation status, resemble those of TPO present in immortalized normal thyrocytes (NTHY cells). Moreover, we found that the peroxidase activity was below the detection threshold of the used methods as a result of a low protein delivery to the cell surface. The differences in post-translational modifications between breast and thyroid TPO are rather minor, which may be explained by a retained antigenicity of breast-expressed TPO shown here for the mammary-derived cell lines and previously demonstrated for TPO expressed in breast tissue [25]. The interaction with TPO-specific antibodies which bind to conformational epitopes strongly depends on intact tree-dimensional structure of the thyroid peroxidase. Our study shows that TPO expressed in breast tissue not only shares similarities with thyroid TPO in biochemical properties (with some minor differences), but also immunodominant regions A and B of breast TPO are specifically recognized by TPOAbs. Therefore, the similarities in biochemical and antigenic properties of breast and thyroid TPO may partially explain the protective role of TPO autoantibodies in breast cancer patients and help further elucidate its mechanism. Future studies should investigate the potential immunotherapies directed against breast-expressed TPO and its specific epitopes.

## Supporting information

S1 Fig Expression of the TPO protein in breast-derived cell lines. **(A) TPO protein expression levels at the indicated time points and (B) its isoelectric point in the analyzed cell lines (B).** Normal human thyroid cell line, NTHY, was used as a positive control. (A) TPO was detected with the ab76935 antibody using Western blotting. 20 μg of total protein lysate were loaded on an 8% SDS-polyacrylamide gel. A β-actin-specific antibody was used as a loading control. (B) 100 μg of total protein lysate was subjected to a two-dimensional (2-DE) electrophoresis. TPO was detected with the ab76935 antibody. NTHY: NTHY-ori 3–1 cell line; pI: isoelectric point.
(TIF)
