# Supplementary figures and images for: Biochemical properties of thyroid peroxidase (TPO) expressed in human breast and mammary-derived cell lines
Source: PLoS One. 2018 Mar 7;13(3):e0193624. doi: 10.1371/journal.pone.0193624 (PMC5841765; doi:10.1371/journal.pone.0193624)

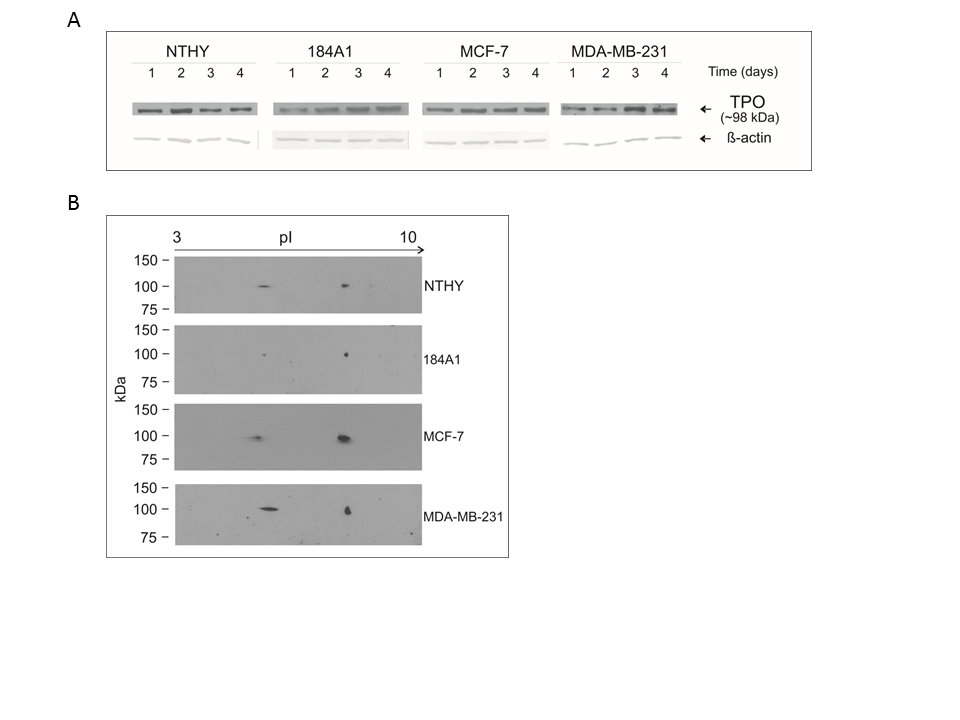

Supplement: S1 Fig — (A) TPO protein expression levels at the indicated time points and (B) its isoelectric point in the analyzed cell lines (B). Normal human thyroid cell line, NTHY, was used as a positive control. (A) TPO was detected with the ab76935 antibody using Western blotting. 20 μg of total protein lysate were loaded on an 8% SDS-polyacrylamide gel. A β-actin-specific antibody was used as a loading control. (B) 100 μg of total protein lysate was subjected to a two-dimensional (2-DE) electrophoresis. TPO was detected with the ab76935 antibody. NTHY: NTHY-ori 3–1 cell line; pI: isoelectric point. (TIF) [file pone.0193624.s001.tif]
